# Perception of the health surveillance users on the health electronic surveillance network (HESN), Saudi Arabia, 2016

**DOI:** 10.1186/s42506-021-00074-1

**Published:** 2021-06-16

**Authors:** Zayid K. Almayahi, Fahad Alswaidi, Abdullah Alzahrani

**Affiliations:** 1 Field Epidemiology Training Program, Ministry of Health, Riyadh, Saudi Arabia; 2 P.O. Box 543, P.C 329 Rustaq, South Batinah Oman; 3 Surveillance and Data Management Unit, Ministry of Health, Public Health HQs, Riyadh, Saudi Arabia; 4 Health Electronic Surveillance Network (HESN), Public Health HQ, Ministry of Health, Riyadh, Saudi Arabia

## Abstract

**Background:**

The established aim of the Saudi Health Electronic Surveillance Network (HESN) is to support the prevention and control of different health events, and to facilitate the delivery of other public health programs. This study aims to evaluate the perceptions of active HESN users regarding its general performance through five major components: practicability, design, data and communication, technical support, and general impression.

**Methods:**

A cross-sectional study was conducted in 2016 using a sample of active HESN users. Out of 1535 active users, 700 were randomly selected. A predesigned electronic questionnaire was sent to each participant via email which was completed by 485 participants. Different composite scores were calculated and compared to the sociodemographic and other technical variables.

**Results:**

The mean age of the participants was 36.92 ± 9.12 (24–65 years), and 57.8% of the sample were male. Riyadh and the KSA’s eastern province represented the highest two regions of participation, at (18.4%) and (14.2%) participants, respectively. About 70.8% were generally satisfied with HESN, while 86.6%% believed that it is better than the traditional paper-work system. Participants who used to work more frequently expressed more level of satisfaction compared to those with minimal use per week or month (*P* ≤ 0.001). Internet speed displayed a significant association with the general level of satisfaction with HESN (*P* < 0.001). Additionally, users who accessed HESN with the Google Chrome browser displayed higher levels of satisfaction when compared to users who relied on other browsers (*P* = 0.003).

**Conclusion:**

Presently, the level of user satisfaction with HESN is reasonable. However, to achieve optimal outcomes for HESN usage, improvements should be considered.

## Introduction

In recent years, countries around the world have started to recognize the essential role played by effective electronic health surveillance and reporting systems for public health. Hence, practitioners, policymakers, and other relevant stakeholders are becoming fully aware of the urgent need for the implementation of such systems [1].

Over the last two decades, this international concern has primarily arisen in response to the increasing prevalence of emerging and re-emerging diseases. Many of these conditions have the potential to cross borders rapidly, with prominent examples including severe acute respiratory syndrome (SARS), the Ebola virus disease (EVD), Middle East respiratory syndrome (MERS), Cholera, and the Zika virus disease [2–5].

In addition to the fact that the traditional surveillance systems are out-of-date, fragmented, non-standardized, and ineffectively integrated into epidemiologic functions, the limited resources and infrastructures of many countries have meant that improvements to novel electronic health surveillance systems are slow, and the literature is still deficient [1, 6]. However, such systems are anticipated to become one of the major and necessary components of public health in the near future.

The Health Electronic Surveillance Network (HESN) was introduced into the Kingdom of Saudi Arabia’s (KSA) public health services to fortify its ability to cope with ongoing global health challenges, and alongside this, to support national health security. It has achieved this by aiding in monitoring disease trends over extended periods of time, generating hypotheses, and detecting clusters and outbreaks in a timely manner. In addition, HESN is expected to aid the country’s public health services in combating biological terrorism, health threats, and facilitating other health programs (e.g., immunization and general health research).

HESN relies on strong and immediate communications among frontline users in designated health facilities, headquarters specialists and health officers, and the central leadership in the Riyadh-based Ministry of Health (MOH) under the Deputy Ministry for Public Health. As an initial stage, HESN has been implemented in the KSA to manage individual cases, outbreaks, immunizations, and vaccine inventories. Many useful tools are integrated into HESN to assist health professionals in monitoring, managing, and reporting on public health issues [7].

At the global level, HESN continues to evolve and remain innovative. Noteworthily, relatively few countries have yet to make this significant and rewarding step [8]. Establishing a solid and successful electronic health system does not simply mean adding or upgrading to a new technical function; rather, it is preceded by a long series of preparatory stages [8]. This involves the availability of sufficient and advanced infrastructure, trained human capital in the field of public health, the ability to devise sensitive and specific algorithms, and the ability to secure sustainable resources and funds [9, 10]. This study aims to evaluate the perceptions of active users of HESN in regards to its general performance through five major components: practicability, design, data and communication, technical support and general impression.

## Methods

This newly developed surveillance system was initially developed by the International Business Machines Corporation (IBM) and used in Canada for the first time under the name of “Panorama.” Saudi Arabia was granted the license to operate it under the name of HESN in 2012 [11, 12].

HESN is a web-based surveillance system connected to all health facilities, where every user has login data. The user is given an authority level based on his or her position. It has bilingual interfaces; Arabic and English. The user chooses one of the six components to use; investigation, outbreak, notification, immunization, work management, and reporting.

### The flow of information of HESN

**Fig. 1:**
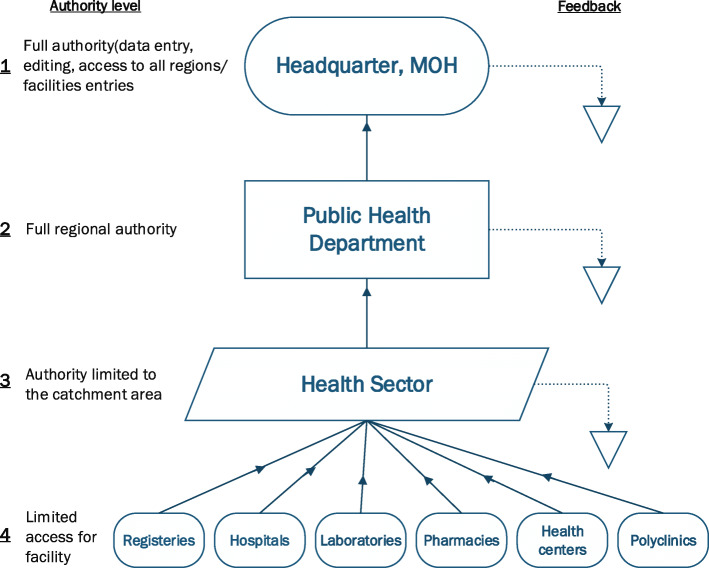
Flowchart for information through the Health Electronic Surveillance Network (HESN) with regards to the authority level and the feedback process

The governmental, nongovernmental, and military health care facilities, either primary, secondary, or tertiary are obliged at this stage to use HESN to notify for the communicable diseases, and also to complete the data for immunization. The data are validated by the health sector (the preventive medicine office serving a specified catchment area within each region), and then the public health department on the respective region, before it reaches to the headquarter in the MOH as demonstrated in Fig. 1. Public health officers working in the health sectors and public health departments are also able to modify, add, or delete the data especially during the cases investigation of the communicable disease. The staff working as the end users in the health care facilities could have medical or paramedical background, and could also be qualified as public health professionals; however, those working in the health sector and public health department are mainly public health oriented.

HESN implementation was started in selected health directorates (Makkah, Qunfudha, Taif, and Jeddah) during the second half of the year 2012, whereas Al-Ahsa and Najran were included in the next year. However, the entire rollout for the remaining 14 health directorates was finished on June 2014. Each implementation stage was preceded by conducting a full infrastructure assessment and enhancement specifically for the availability of computers and internet connection, and also by conducting a full customized training to the staff. Selected staff from each health directorate were trained by experts of HESN from the central unit in MOH, as well as by staff from other regions who were qualified as trainers. Each training course took about 1 month, and was performed in three stages. First stage was to train the staff about all components and the full usage of HESN, whereas the second stage was to let the trainees use HESN practically under the supervision. The qualified trainees from each health directorate were then providing the training to the end users from all health facilities in the same region as a third stage.

### Research setting

A list of all HESN users was obtained from the Deputy Ministry for Public Health’s HESN unit. Users were from different areas in the health sector, both governmental and private. These included the MOH’s HESN unit, designated health directorates, and hospitals and primary health care centres (PHCCs) around the KSA. Additionally, the study took place between June 2016 and July 2016.

### Target population

Target population were all registered HESN users affiliated to governmental or nongovernmental institutions (*N* = 11,324).

### Study population

The study population was comprised of all HESN users with valid email contacts, and who had previously used their HESN account at least once for either immunization or investigation purposes. The total number of users who met the inclusion criteria were 1535 (14%). This means that there was a significant number of users whose emails were missing, in addition to others where the role was only limited to supervision and analysis, and not actively entering the data as the case of the end users who are the most important keys of HESN. Other people may have changed their duties and works, and thus their accounts were dormant.

### Design

The research design utilized for this study was a cross-sectional design.

### Sample size

Assuming 50% satisfaction with HESN functionality and performance at 95% confidence interval, the required sample size was 384, using Epi Info™ version 7 (CDC, Atlanta, GA, USA). A list of all users with valid emails (*n* = 1535) was generated, and in turn, a simple random sampling technique was applied to identify the required number.

Upon the trial done to ensure good understanding of the questionnaires, and also to estimate the response, we found errors in some of the emails. Therefore, to ensure the required response rate and in order to compensate for the invalid emails and incomplete answers, the decision was made to distribute the questionnaire to 700 randomly selected HESN users.

### Data collection

The predesigned questionnaire was developed by the researcher and reviewed by the central unit department in MOH, after careful understanding to all the different components of HESN including the type of information needed to be entered and analyzed, the way of input and output of the data, and the different setup required for successful use. The questionnaire was created using the “Survey Monkey” website, and in turn, emailed to all participants.

The questionnaire included two categories: firstly, a category collecting data pertaining to the participants’ demographic characteristics, technical knowledge, and surveillance experiences. Secondly, a category collecting data pertaining to the users’ perceptions regarding the overall use and design of HESN through 40 questions, which were used to generate five different composite scores. Each composite score was computed for each participant by taking the mean of a number of questions. The mean and standard deviation (SD) were then calculated for each composite score independently.

Scores of 11 questions on performance and daily practice were used to obtain a composite practicability score and similarly, a composite score for perceptions to HESN design was calculated by using 5 questions (user interface, and data output). The third, fourth, and fifth composite scores on communication and completeness of data, technical support, and general impression were calculated using 11 (communications/updates, laboratory information, accuracy of data, and search), 7 (technical support and logistic support), and 6 questions (impression and satisfaction), respectively.

Specifically, a 5-point Likert scale was used to gauge participants’ opinions which utilized a spectrum of responses as follows: “Strongly Disagree,” “Disagree,” “Neutral,” “Agree,” and “Strongly Agree.” They were coded as 1, 2, 3, 4, and 5 respectively.

All five sections of the questionnaire obtained a Cronbach’s alpha (α) above the acceptable standard value of 0.7; practicability 0.899, design 0.844, data completeness and communication 0.91, technical support 0.774, and general impression 0.836. The total Cronbach’s alpha coefficient for internal consistency was 0.961.

The questionnaire was emailed to 700 participants on 2 June 2016, and subsequent reminders were sent periodically. The questionnaire was designed bilingually using Arabic and English, and the choice as to which language to use in completing the questionnaire was left to each participant’s preferences. The questionnaire was first developed in English by reviewing available surveys and experiences in the literature [1, 12], but it was tailored for the HESN setting, and the study objectives. For the translation into Arabic, a native Arabic speaker and Arabic bilingual expert in public health and epidemiology carried out the translation from English into Arabic. The questionnaire’s content was then reviewed by two public health experts with HESN experience and face-validated by piloting it among 10 public health professionals before the study for doubtful or confusing items. The total number of responses was 607 (response rate = 87%), and since a form was only considered complete if the participant validly answered at least 90% of the questions, the completed forms amounted to 80% (*n* = 485).

### Statistical analysis

IBM SPSS 21.0 (IBM, Armonk, NY, USA) was used to organize, tabulate, and statistically analyze the data. Numerical data were presented as means and standard deviations, whereas numbers and percentages were used to present categorical data. Normality assumption was evaluated with Kolmogorov-Smirnov test. Mann-Whitney *U* test and Kruskal-Wallis test were used, as appropriate, to compare groups in terms of different composite scores, while the *P* value was considered statistically significant if < 0.05

### Ethical considerations

This research was revised by the Saudi Field Epidemiology Training Program (FETP; Centers for Disease Control and Prevention [CDC]; Atlanta, Georgia USA) scientific board, as was accepted technically and ethically. In order to contribute to the research by completing the questionnaire, all participants were asked to complete a written consent form. In addition, data were collected anonymously and used only for the purposes of the study. Finally, data confidentiality was assured throughout the whole study.

## Results

Table 1 describes the demographic characteristics of the study participants in relation to the composites scores of practicability, design, data and communication, technical support, and general impression. The mean and SD for the age variable was 36.92 ± 9.12, with a range from 24 to 65 years. Approximately, two-thirds of the participants (68%) were aged 30–50 years, and males represented higher proportion than females (57.3%). The participants were mainly Saudis (43.9%), and the remainder were divided into two groups: firstly, Arabs (27.2%); and secondly, non-Arabs (28.9%). Almost 77% of participants had a graduate degree, compared to 23% with post graduate degree such as master, PhD, or residency programs.

**Table 1:** Characteristics of the study participants in relation to the composites scores of practicability, design, data and communication, technical support, and general impression, KSA, 2016

Socio-demographic	*n*	Composite practicability score	Composite design score	Composite data and communication score	Composite technical support scale	Composite impression scale
Age						
< 30	107	3.71 ± 0.69	3.74 ± 0.77	3.63 ± 0.67	3.34 ± 0.74	4.00 ± 0.69
30–50	330	3.62 ± 0.69	3.58 ± 0.74	3.55 ± 0.69	3.28 ± 0.72	3.96 ± 0.71
> 50	48	3.65 ± 0.60	3.34 ± 0.74	3.60 ± 0.54	3.25 ± 0.57	3.98 ± 0.65
*p* value†		0.792	0.045*	0.909	0.642	0.726
Gender						
Male	278	3.57 ± 0.73	3.54 ± 0.78	3.50 ± 0.71	3.23 ± 0.74	3.95 ± 0.73
Female	207	3.74 ± 0.61	3.76 ± 0.65	3.68 ± 0.60	3.37 ± 0.66	3.99 ± 0.66
*p* value!		0.031*	0.002*	0.003*	0.045*	0.173
Nationality						
Saudi	213	3.56 ± 0.75	3.50 ± 0.84	3.49 ± 0.75	3.11 ± 0.77	3.93 ± 0.79
Arab	132	3.60 ± 0.60	3.63 ± 0.65	3.53 ± 0.60	3.34 ± 0.63	4.00 ± 0.65
Non-Arab	140	3.81 ± 0.62	3.84 ± 0.56	3.74 ± 0.57	3.52 ± 0.62	4.00 ± 0.59
*p* value†		0.006*	0.001*	< 0.001*	< 0.001*	0.587
Education						
Graduate degree	372	3.68 ± 0.70	3.66 ± 0.75	3.62 ± 0.68	3.29 ± 0.73	3.99 ± 0.71
Post graduate	113	3.51 ± 0.63	3.53 ± 0.67	3.44 ± 0.60	3.29 ± 0.67	3.90 ± 0.65
*p* value!		0.014*	0.038*	0.006*	0.984	0.015
Current work specialty						
Administration	106	3.70 ± 0.67	3.68 ± 0.64	3.59 ± 0.61	3.37 ± 0.66	3.96 ± 0.73
Clinical	141	3.66 ± 0.65	3.73 ± 0.64	3.65 ± 0.58	3.35 ± 0.67	3.92 ± 0.61
Public health	238	3.60 ± 0.71	3.55 ± 0.81	3.52 ± 0.74	3.21 ± 0.76	4.00 ± 0.73
*p* value†		0.662	0.118	0.227	0.133	0.516
Surveillance experience						
Yes	323	3.66 ± 0.68	3.64 ± 0.75	3.58 ± 0.68	3.30 ± 0.70	3.98 ± 0.71
No	162	3.61 ± 0.69	3.62 ± 0.70	3.56 ± 0.65	3.26 ± ± 0.73	3.95 ± 0.68
*p* value!		0.359	0.642	0.457	0.449	0.458
Electronic surveillance						
Yes	115	3.59 ± 0.77	3.53 ± 0.83	3.51 ± 0.77	3.35 ± 0.65	3.88 ± 0.76
No	370	3.66 ± 0.65	3.66 ± 0.70	3.60 ± 0.63	3.27 ± 0.73	4.00 ± 0.68
*p* value!		0.68	0.195	0.375	0.291	0.134
Use frequency						
More frequently per day/week	396	3.72 ± 0.64	3.68 ± 0.71	3.63 ± 0.66	3.33 ± 0.7	3.86 ± .68
Once a week or month	89	3.3 ± 0.76	3.4 ± 0.78	3.35 ± 0.66	3.1 ± 0.72	3.57 ± 0.71
*p* value!		< 0.001*	0.003*	< 0.001*	0.015*	< 0.001*

*P* < 0.05.! Mann-Whitney, †Kruskal-Wallis tests, * statistically significant

Riyadh and the KSA’s eastern province represented the two highest regions of participation (18.4%) and (14.2%) participants, respectively. Regarding the work specialty, approximately half of the participants were working in public health activities (49.1%), while participants involved in clinical works comprised about (29.1%). Almost two-thirds of participants (66.6%) had already experienced working in the public health surveillance system, whereas only (23.7%) had previously worked with electronic medical records.

Notably, the estimated composite scores of HESN users were significantly associated with gender, nationality, educational level, and use frequency. The level of satisfaction among female users were greater than male users regarding most of studied aspects particularly for the composite design score (*P* = 0.002), and likewise among Non-Arab users who were more satisfied than both Arabs and Saudis (*P* = 0.001). Surprisingly, users with post graduate degree showed less agreement to the different aspects of HESN functionality in comparison to those with graduate degree and likewise for the general impression composite score (*P* = 0.015). Participants who used to work on HESN more frequently expressed more level of satisfaction compared to those with minimal use per week or month (*P* ≤ 0.001). Unexpectedly, the surveillance work experience and the current work specialty had no significant relationship.

Table 2 describes the different technical and functional aspects of HESN in respect to the composites scores of practicability, design, data and communication, technical support, and general impression. Most of the participants (85.8%) underwent training, while the remainder (14.2%) did not. Of the participants who underwent training, less than half (46.4%) received training once only.

**Table 2:** Functional and operational aspects of HESN in respect to the composites scores of practicability, design, data and communication, technical support, and general impression, KSA, 2016

Technical aspects	*n*	Composite practicability score	Composite design score	Composite data and communication score	Composite technical support scale	Composite impression scale
Training						
Once	225	3.62 ± 0.70	3.65 ± 0.72	3.56 ± 0.66	3.24 ± 0.71	3.96 ± 0.71
More than once	191	3.67 ± 0.70	3.61 ± 0.77	3.60 ± 0.69	3.34 ± 0.71	3.99 ± 0.69
No training	69	3.64 ± 0.59	3.62 ± 0.68	3.58 ± 0.62	3.30 ± 0.74	3.93 ± 0.70
p-value†		0.702	0.909	0.573	0.307	0.855
Internet speed						
Fast	94	3.98 ± 0.62	3.87 ± 0.65	3.80 ± 0.58	3.65 ± 0.61	4.22 ± 0.56
Moderate	239	3.74 ± 0.59	3.71 ± 0.66	3.64 ± 0.61	3.38 ± 0.64	4.03 ± 0.66
Slow	152	3.28 ± 0.70	3.36 ± 0.80	3.33 ± 0.74	2.91 ± 0.72	3.72 ± 0.76
*p* value†		< 0.001*	< 0.001*	< 0.001*	< 0.001*	< 0.001*
Internet connection						
Home net	30	3.75 ± 0.79	3.76 ± 0.86	3.72 ± 0.78	3.21 ± 0.90	4.00 ± 0.87
Work and home net	169	3.74 ± 0.63	3.72 ± 0.67	3.64 ± 0.62	3.30 ± 0.74	4.09 ± 0.64
Work net	286	3.58 ± 0.70	3.56 ± 0.75	3.52 ± 0.68	3.29 ± 0.67	3.89 ± 0.70
*p* value†		0.133	0.074	0.294	0.744	0.088
Authority						
Entering and presenting data	209	3.60 ± 0.66	3.58 ± 0.73	3.55 ± 0.66	3.18 ± 0.69	3.96 ± 0.68
Full use	103	3.68 ± 0.70	3.7 ± 0.70	3.60 ± 0.71	3.31 ± 0.78	4.08 ± 0.72
Only entering data	173	3.67 ± 0.71	3.66 ± 0.74	3.60 ± 0.65	3.41 ± 0.69	3.92 ± 0.70
*p* value†		0.397	0.352	0.732	0.009*	0.223
Function						
Immunization	79	3.67 ± 0.70	3.72 ± 0.78	3.51 ± 0.66	3.10 ± 0.78	3.91 ± 0.89
Investigation	101	3.68 ± 0.66	3.64 ± 0.66	3.63 ± 0.64	3.38 ± 0.64	4.06 ± 0.66
Notifications management	53	3.64 ± 0.74	3.61 ± 0.75	3.67 ± 0.63	3.33 ± 0.80	4.01 ± 0.66
Outbreak investigation	141	3.56 ± 0.75	3.53 ± 0.82	3.52 ± 0.75	3.17 ± 0.74	3.91 ± 0.73
Reporting	100	3.72 ± 0.59	3.73 ± 0.62	3.62 ± 0.59	3.50 ± 0.54	3.98 ± 0.55
Work management	11	3.55 ± 0.55	3.47 ± 0.57	3.42 ± 0.62	3.14 ± 0.81	4.05 ± 0.53
*p* value†		0.840	0.178	0.429	<0.001*	0.684
Internet browser						
Firefox	235	3.62 ± 0.69	3.60 ± 0.76	3.53 ± 0.65	3.24 ± 0.72	3.97 ± 0.71
Google chrome	222	3.72 ± 0.65	3.72 ± 0.66	3.66 ± 0.67	3.36 ± 0.70	4.01 ± 0.67
Internet explorer	28	3.25 ± 0.75	3.25 ± 0.86	3.29 ± 0.67	3.12 ± 0.69	3.57 ± 0.73
*p* value†		0.003*	0.011*	0.005*	0.08	0.003*
Interface language						
Arabic	159	3.59 ± 0.68	3.56 ± 0.74	3.51 ± 0.65	3.16 ± 0.74	3.93 ± 0.77
English	209	3.71 ± 0.66	3.71 ± 0.67	3.65 ± 0.64	3.44 ± 0.66	3.97 ± 0.61
Both Arabic and English	117	3.6 ± 0.72	3.59 ± 0.81	3.54 ± 0.73	3.6 ± 0.72	4.01 ± 0.74
*p* value		0.355	0.183	0.063	<0.001*	0.568

*P*< 0.05, †Kruskal Wallis tests, * statistically significant

Firefox and Google Chrome were the most commonly used Internet browsers (48.5% and 45.8%, respectively). Internet speed was classified as “Moderate” by nearly half of the participants (49.3%), compared to 19.4% who classified this variable as “Fast.” Majority (59%) used the internet connection of work to open HESN, (6.2%) used home connection while the remaining (34.8%) used work and home connections too. The English interface was used by (43.1%) participants, and Arabic interface by (32.8%), while (24.1%) used both the Arabic and English interfaces.

Remarkably, users with a fast Internet speed were more satisfied than those with a moderate and slow Internet speed for all composite scores (*P* < 0.001). Similarly, participants who used HESN with the Google Chrome browser were more satisfied, compared to users of Firefox and Internet Explorer. The authority level of the users, the interface language used, and the different functions of HESN only showed significant relation with the composite score of technical support (*P* = 0.009, 0.001, < 0.001) respectively. However, the type of internet connection, and the training frequency did not show any significant differences with any of the calculated composite scores.

**Fig. 2:**
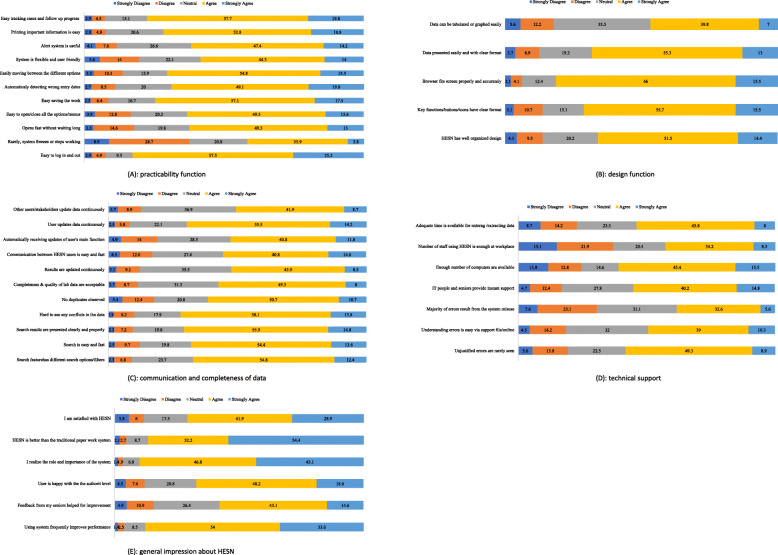
User’s perception and experience regarding the functionality of HESN. A Practicability, B Design, C Communication and completeness of data, D Technical support, and E General impression

Figure 2a–e shows the data related to users’ perceptions and experience regarding the different functionality of HESN. Approximately 41.7% of the participants agreed that HESN rarely freezes or stops working suddenly. Almost (58.3%) of participants agreed that HESN is a flexible and user-friendly application, compared to (19.6%) who disagreed and (22.1%) who responded neutrally. Approximately two-thirds (65.9%) believed that it has a well-organized design, and furthermore, that its key functions or buttons have a clear format and specific order. The majority (68.0%) agreed that HESN is equipped with an easy and fast search system, while 61.4% stated that they had not encountered any duplicates. Only 57.3% showed satisfaction on laboratory data completeness and quality, compared to 11.4% who were not, while 31.3% remained neutral. Finally, more than two-thirds (70.8%) were generally satisfied with HESN, and 86.6%% believed that HESN is better than the traditional paper work system.

## Discussion

There is a limited number of papers in the literature which have described the use or the advantages of using the electronic health records for public health surveillance. However, the several experiences in the clinical settings in some countries suggest that the electronic health surveillance systems have great promise to improve the public health surveillance and the outcome.

Accordingly, it is critical to assess health surveillance systems periodically, and this is even more critical in the case of electronic health surveillance systems. Furthermore, user perceptions are crucial to consider in order to ensure the success of public health systems.

The implementation of HESN was started in the mid of 2012, before few months of the official announcement of the emergence of a novel coronavirus (MERS-CoV) in the KSA [13]. Intuitively, the several outbreaks of MERS-CoV in the kingdom provided opportunity to practically use and assess HESN as an early notifying surveillance system. Moreover, until the full implementation which was accomplished in the mid of 2014, other unpredicted infectious diseases had re-emerged globally such as Ebola. This indicates that the current time is crucial to develop intelligent surveillance systems where the periodic assessment, evaluation, and updating are continuously needed. The randomly selected sample of HESN users represented a fairly unbiased assortment of individuals with varying backgrounds and characteristics. Notably, all regions and cities of the KSA were represented in the sample.

In general, the results showed a reasonable level of satisfaction (70.8%) among HESN users. However, their satisfaction was remarkably associated with the following factors: Internet browser, Internet speed, use frequency, gender, nationality, and education. Other factors had minimal significant association including; age, interface language, different functions of HESN, authority level, and the type Internet connection used.

***The role of internet speed and internet browser:***

The successful adoption of any electronic health system relies mainly on the degree to which the infrastructure setup has been prepared effectively [14]. In the present study, users who rated their Internet speed as fast represented less than 20% of the sample. It is noteworthy, then, that a significant number of users employed HESN at home because the speed of their Internet connection at work was slow. This indicates that the infrastructure has yet to be improved, particularly with respect to the variable of the stability of the Internet connection. The same challenges have been encountered after establishing electronic health surveillance systems in China, where the evidence indicates that professionals have employed their mobile phones to overcome the unavailability of computers and the Internet [15–17]. In the case of HESN users with a reasonable internet speed, they tended to be more satisfied and recommend more improvements. Generally, therefore, it is possible to conclude that the unstable Internet connections are one of the major difficulties professionals encounter when working with electronic systems, especially in rural areas where infrastructure continues to be deficient [18].

At the same time, it is important to gain insight into the type of the Internet browser employed by HESN users, since certain key functions or software extensions may only be compatible with one browser but not others. Noticeably, the results indicated that the most satisfying browser for the present study’s sample was Google Chrome, followed by Firefox and Internet Explorer.

***Multi-purpose system:***

HESN is a multi-functional system which serves different objectives. The results indicated that investigation purposes form the core function of the system, which do require extensive data and communications to be completed quickly. Users involved in the outbreak investigation function showed less satisfaction with regards to the technical support provided, which somehow reflect the complexity of work in such cases. It can also be explained by inadequacy of staff and or limit of the time; only 42.7% and 53.8% of respondents agreed that staff and time were adequate respectively. This goes in consistence with previous studies that have identified different challenges with electronic public health surveillance systems in Peru. Other factors which affect outbreak investigations using electronic surveillance systems are potentially related to the unnecessary time delay, inadequate sensitivity, poor positive predictive value, the significant turnover of trained personnel, and poor data quality [19].

***Authority level and the software’s used language:***

HESN users had different levels of authority, but this variable was insignificantly related to their perceptions of the system, except for the technical support aspect. Noteworthily, as indicated by the existing literature, this was not the case with other electronic systems, with the results suggesting that perceptional differences exist between basic and advanced users [20].

The majority of respondents considered HESN a user-friendly software. However, the results showed that users of the English interface showed more satisfaction of the system compared to Arabic interface users, though this only showed significance with technical support aspect. Likewise, non-Arab users showed more noteworthy satisfaction compared to Saudis and Arabs. This is indicative of the fact that the Arabic coding of different software and systems may be troublesome or perhaps that Arabic translation of the functions may not be appropriate. Indeed, HESN is a developed version of the Canadian “Panorama” software, which was built primarily using the English language [11, 12]. The high satisfaction level toward HESN among the non-Arab users may also be attributed to the use of the English interface. Notably, one of the major reasons why the electronic surveillance system for malaria in Thailand succeeded is due to the functional design of the system, which provided malaria staff with close-to-real-time case management data quality [18].

***The role of usage:***

Clear differences were observed in the frequencies HESN usage, and furthermore, the results indicated that these differences significantly affected the participants’ impressions of the system. One way in which to account for this result is by stating that when users interacted with HESN more frequently, they were exposed to different technical issues, they learned to manage them, and they started to locate the correct keys and buttons rapidly. Subsequently, they saved time due to their acquired competency, thus entering a position where they could recommend further improvements to the system.

It is always fundamental to study and understand the setting and context carefully before designing any new system. This is because the findings from such inquiries can ensure effective model processes, with which obstacles can be overcome when using electronic health data [21]. Many users agreed that freezing is not an uncommon issue, and so the encountered errors may be the result of technical defects within the system rather than misuse. Therefore, a thorough technical evaluation to the system may be needed.

A significant proportion of HESN users also found that tabulation and graphing of data is not a straightforward task. Improving this would certainly help users understand their important role, thus ensuring that they participate intellectually. The literature also indicates that even non-specialists can benefit from the availability of automated analysis (specifically, those which generate graphics and tables easily), particularly when performing complex assessments in short periods of time [22].

Although many of the present study’s participants agreed that data conflict and data duplication issues are not common issues with HESN, it is critical to ensure that data are consolidated and not repeated. This is a particularly crucial point with respect to laboratory data, which must be accurate and updated instantly. Noteworthily, conflict or discord issues within data sets have been identified in other studies of electronic surveillance programs [18]. Indeed, the development of electronic health systems should have the advantage of preventing any medical errors, or as an alternative, it should aid in reducing health care disparities [23].

Human errors may also occur, for instance, the transcription of paper-based data to the system. Notably, the incidence of such errors can be limited by using electronic systems for data collection and data entry, provided with logic check programs [18, 24].

Finally, it is critical to recognize that issues such as securing a fast Internet speed are as important as providing an adequate number of working staff. This is because all these factors ensure that the work is being updated and conducted in a suitable manner. Therefore, it is worth emphasizing that a significant proportion of this study’s participants believed that their workplaces were neglecting to draw on adequate levels of human capital.

This study recommends the need for public health electronic surveillance systems especially during this time of frequent epidemics and pandemics. It is essential to ensure the readiness and availability of all aspects of infrastructure setup before anything else, including the accessibility and speed of the internet, while developing the best and friendly design, and interface, and selecting the appropriate browser when applicable. Surveillance users get more satisfaction when they spend more time on using the system which undoubtedly becomes more effective and successful.

### Limitations

Minimizing the collection of certain types of demographic data (e.g., age and gender) may help to improve the quality of data and reduce bias. It is possible that certain participants were not comfortable sharing their opinions freely as a consequence of the focus on demographic variables. Also, this study did not consider performing multivariate analysis to adjust for potential confounders. The use of both Arabic and English questionnaires during the data collection complicated and lengthened the process of logging and analyzing the data. One language would have saved more time. The survey was purposefully developed for HESN with the respect of and based on other literature experiences, though they were few and mostly unvalidated.

## Conclusion

A reasonable level of satisfaction was observed among HESN users in this study. However, to ensure that the HESN’s public health goals are achieved, various important improvements should be considered. As indicated by the results, user satisfaction was significantly affected by the frequency of use, Internet speed, and the type of Internet browser used to access HESN. Therefore, it is clear that the basic infrastructure requirements must be established adequately, particularly regarding the issues of Internet connection, computers, and staff. Further studies to explore the effectiveness of HESN as a public health surveillance are unquestionably required.

## Abbreviations

HESN: Health Electronic Surveillance Network
KSA: Kingdom of Saudi Arabia
SARS: Severe acute respiratory syndrome
EVD: Ebola virus disease
MERS: Middle East respiratory syndrome
MOH: Ministry of Health
PHCCs: Primary health care centres
SD: Standard deviation
